# Artemisinin derivative DHA27 enhances the antibacterial effect of aminoglycosides against *Pseudomonas aeruginosa* by inhibiting mRNA expression of aminoglycoside-modifying enzymes

**DOI:** 10.3389/fphar.2022.970400

**Published:** 2022-10-24

**Authors:** Nuoyan Wang, Xuemin Chen, Jing Luo, Fei Deng, Fuguo Shi, Qin Wu, Yasi Huang, Qin Ouyang, Rongxin Qin, Hong Zhou

**Affiliations:** ^1^ Key Laboratory of Basic Pharmacology, Ministry of Education and Joint Laboratory of International Cooperation, Ministry of Education of Characteristic Ethnic Medicine, College of Pharmacy, Zunyi Medical University, Zunyi, China; ^2^ Department of Pharmaceutical Chemistry, College of Pharmacy, Army Medical University (The Third Military Medical University), Chongqing, China; ^3^ Department of Pharmacology, College of Pharmacy, Army Medical University (The Third Military Medical University), Chongqing, China

**Keywords:** DHA27, tobramycin, *Pseudomonas aeruginosa*, antibacterial sensitizer, antibiotic resistance, aminoglycoside-modifying enzymes

## Abstract

Bacterial resistance is becoming increasingly serious, the present study aimed to investigate the mechanism of antibacterial sensitization effect of DHA27 combined with tobramycin in tobramycin-resistant *Pseudomonas aeruginosa* (PA). We found that DHA27 combined with aminoglycosides had an antibacterial sensitization effect on PA. Tobramycin, owing to its lower toxic and side effects, was selected to further study the molecular mechanism of drug combination. A sublethal-dose bacterial challenge/sepsis mouse model was established to study the protective effect of DHA27 plus tobramycin. Scanning electron microscopy was used to investigate whether DHA27 exerts the antibacterial sensitization effect by directly affecting bacterial morphology. The effect of DHA27 on daunorubicin accumulation in bacteria was studied, and quantitative reverse transcription PCR was used to study the effect of DHA27 plus tobramycin on 16S rRNA methyltransferase and aminoglycoside-modifying enzyme mRNA expression. Twenty clinical isolates of PA were found to be tobramycin resistant; DHA27 plus tobramycin had a significant antibacterial sensitization effect on many of these resistant strains. DHA27 plus tobramycin reduced the bacterial load in the spleen and lungs of sepsis model mice and levels of proinflammatory cytokines interleukin-1β (IL-1β) and interferon-γ (IFN-γ). DHA27 plus tobramycin significantly inhibited the mRNA expression of aminoglycoside-modifying enzymes in bacteria. DHA27 combined with AGs had an antibacterial sensitization effect on PA; the molecular mechanism underlying this effect is closely related to the inhibition of the mRNA expression of aminoglycoside-modifying enzymes, especially *aac(3)-II*.

## 1 Introduction

Since the discovery and application of antibiotics in the treatment of bacterial infections, numerous antibiotics have been discovered and used in clinical practice, resulting in increasingly serious bacterial resistance and difficulties in developing new antibiotics (Hutchings et al., 2019). The emergence of bacterial resistance increases the risk of infection, prolongs the time of diagnosis and treatment, and aggravates the symptoms of patients. Infections caused by resistant strains are associated with higher diagnostic and treatment costs and patient mortality than those caused by susceptible strains (Naylor et al., 2018; Zhen et al., 2019). According to the Centers for Disease Control and Prevention report from 2019, annually, more than 2.8 million people in the United States are infected with drug-resistant bacteria, and more than 35,000 people die from them (“Antibiotic resistance threats in the United States, 2019,” 2019). The U.S. Food and Drug Administration (FDA) approval rate for antibiotics and the number of approved antibiotics have been on the rise since 2019 (Andrei et al., 2019). The rate of development and application of new antibiotics is lower compared with the rate of bacterial resistance development, and the large number of pan-resistant and fully resistant bacteria has made the problem worse (Zaman et al., 2017). Therefore, all countries are committed to finding a breakthrough to treat bacterial infections.

According to the latest analysis by the China antimicrobial surveillance network (CHINET), currently, most clinically isolated bacteria are Gram negative, with an isolation rate of 71.9%. *Pseudomonas aeruginosa* (PA) has the highest isolation rate among non-fermenting Gram-negative bacilli (Hu et al., 2021). The resistance to commonly used drugs such as β-lactams, aminoglycosides (AGs), and fluoroquinolones has also increasing in the clinical treatment of PA infections (Zhang et al.).

As an opportunistic pathogen, PA is the leading cause of infection and death in patients with cystic fibrosis and immunocompromised patients, and because of its significant drug resistance, it has become increasingly challenging to cure infections caused by PA (Pang et al., 2019). In addition, the overuse of antimicrobials accelerates the development of multidrug-resistant PA strains, leading to a gradual loss of efficacy of empirical antimicrobial therapy (Hirsch and Tam, 2010). The antibiotic resistance mechanisms of PA are mainly of three types: intrinsic, acquired, and adaptive. The intrinsic resistance mechanisms include production of antibiotic-modifying enzymes, gene expression of an active efflux pump, and reduced permeability of bacterial outer membrane (Pang et al., 2019). Acquired resistance may be achieved through the transfer of resistance genes between bacteria and genetic mutation (Breidenstein et al., 2011). Adaptive resistance is a transient change in genes and/or proteins caused by environmental stimuli that increase the viability of bacteria, and the resistance may be reversed when the stimulus is removed (Sandoval-Motta and Aldana, 2016). The most significant feature of adaptive resistance in PA results in infection of patients with cystic fibrosis (CF) and poor prognosis of biofilm formation and generation of persistent cells (Taylor et al., 2014).

Our research group has been conducting long-term studies on the antibacterial sensitization of artemisinin derivatives. Previous studies have found that artemisinin derivative DHA27 combined with β -lactam antibiotics have antibacterial sensitization effect on *E. coli* (Song et al., 2016). However, its antibacterial sensitizing effect has not been studied in other bacteria. Due to the high clinical isolation rate of PA and AG antibiotics are commonly used antibiotics in clinical practice for the treatment of PA infection. In the present study, we investigated the antimicrobial sensitization effect of the artemisinin derivative DHA27, in combination with various AG antibiotics, on clinical isolates of PA and further examined the molecular mechanism of its antibacterial sensitization effect. We found that DHA27, in combination with the AG antibiotic tobramycin, had an antimicrobial sensitization effect on the PA standard strain ATCC27853.

## 2 Materials and methods

### 2.1 Reagents

DHA27 was synthesized by College of Pharmacy, Zunyi Medical University and College of Pharmacy, Army Medical University (the Third Military Medical University) (Song et al., 2016). Gentamicin, tobramycin (TOB), and amikacin were purchased from Beijing Solarbio Science & Technology Co., Ltd. whereas daunorubicin (DNR) was purchased from Med Chem Express (USA). RNA iso Plus and Reverse Transcription Kit were purchased from TaKaRa (Japan). Primer design and synthesis were performed by Sangon Biotech (Shanghai) Co., Ltd. Interleukin (IL)-1β (mouse) and interferon (IFN)-γ (mouse) ELISA kits were purchased from Thermo Fisher Scientific (USA).

### 2.2 Laboratory animals

Specific-pathogen-free (SPF) male BALB/c mice, 6–8 weeks old, weighing 20–22 g, were purchased from Chongqing Labite Biotechnology Co., Ltd (animal license number: SCXK Xiang 2019-0004). The experimental animals were adaptively fed for 1 week in the SPF animal room, with free access to food and water, and maintained at a 12 h light: dark cycle per day at 22°C–24°C and 60 ± 5% humidity. This experiment strictly complied with the humanized use and care guidelines of the American Institute of Health. All protocols and experimental procedures involving live animals were approved by the Animal Care Welfare Committee of GuiZhou Medical University (license number: 1,804,102).

### 2.3 Bacterial strains and culture

PA standard strain ATCC27853 was maintained in our laboratory. Twenty clinical isolates of PA donated by Zunyi Aerospace Hospital in Guizhou were stored at -80°C in our laboratory. The clinical isolates were plated using the streaking method. After incubation at 37°C for 18–24 h, a single colony was picked, placed in 2 ml Mueller-Hinton (MH) broth, and incubated in a shaker at 37°C at 180 rpm for 1–2 h for activation. Then, 10 ml of MH broth was added and the constant temperature shaking culture was continued until the optical density value at 600 nm (OD_600_) was 0.3–0.8.

### 2.4 Drug susceptibility assay

PA Bacteria in the exponential phase of growth (1.0 × 10^6^ colony-forming units (cfu)/mL were inoculated in 96-well plates and incubated at 37°C for 24 h. The minimum inhibitory concentrations (MICs) were determined by serial two-fold dilutions in MH broth containing AGs in accordance with the Clinical and Laboratory Standards Institute (CLSI) guidelines (Humphries et al., 2021). The MIC value was taken as the lowest drug concentration at which inhibition of bacterial growth could be observed. The MICs of drug combinations were also assayed. Whether two drugs had a synergistic effect was determined on the basis of the fractional inhibitory concentration index (FICI) (Odds, 2003), which refers to the sum of the ratios of MICs in the combination of various drugs and the MIC of the single drug when two or more drugs are used at the same time, which is the gold standard for evaluating the combined effect of drugs. In general, FICI ≤0.5 signifies a synergistic effect, and FICI >4.0 indicates an antagonistic effect; FICI between 0.5 and 4.0 is irrelevant (Odds, 2003).

### 2.5 Determination of bacterial load and inflammatory cytokine levels in sublethal dose sepsis model mice

Twenty-eight male BALB/C mice were randomly divided into seven groups, with four mice per group. Mice in each group were administered the best antimicrobial sensitizing effect of the clinical isolate PA16 (3.0 × 10^8^ CFU/kg body weight; according to 0.1 ml/20 g body weight) through the caudal vein. Next, 38.4 mg/kg/d TOB, 20 mg/kg/d DHA27, 30 mg/kg/d DHA27, 38.4 mg/kg/d TOB +20 mg/kg/d DHA27, or 38.4 mg/kg/d TOB +30 mg/kg/d DHA27 was slowly administered through the caudal vein, respectively, according to the treatment group. Mice in the blank control group were administered normal saline (0.1 ml/20 g body weight) through the caudal vein. The first dose was followed by bacterial challenge; the second dose and the third dose were administered 8 h and 16 h after bacterial challenge. After bacterial challenge for 24 h, mice were sacrificed after inhalation anesthesia. After weighing the spleen and lungs tissues, normal saline was added at a ratio of 1:10, with a grinding frequency of 60 Hz and a grinding time of 60 s. The tissues homogenates were diluted 10 times and coated on MH plates. After incubation at 37°C for 18–24 h, the number of colonies was counted to calculate the bacterial load. The levels of proinflammatory cytokines such as IL-1β and IFN-γ in the spleen and lungs tissue homogenates were detected by ELISA.

### 2.6 Scanning electron microscopy

The concentration of the activated bacterial solution was adjusted to 1 × 10^6^ CFU/ml. According to the optimal concentration of DHA27 in combination with TOB to exert the greatest antibacterial sensitization effect, the groups were established as follows: broth group, dimethyl sulfoxide (DMSO) group, 1/16 MIC DHA27 (64 μg/ml) group, 1/16 MIC TOB (64 μg/ml) group, and 1/16 MIC TOB (64 μg/ml) + 1/16 MIC DHA27 (64 μg/ml) group. The bacterial solution was, incubated at 37°C with constant-temperature shaking at 180 rpm for 6–8 h and centrifuged at 537 × *g* for 3 min. The pellet was washed thrice with phosphate-buffered saline (PBS) and resuspended in 0.1 ml of PBS. Then, 10 µl of the bacterial suspension from each group was placed onto a coverslip, and the coverslips were placed in a constant-temperature incubator at 37°C for 30 min. Then, the samples were fixed using a glutaraldehyde fixative solution and sent to the electron microscopy laboratory to observe bacterial morphology with scanning electron microscopy (SEM).

### 2.7 *Daunorubicin accumulation in Pseudomonas aeruginosa*

DNR, an antitumor drug, shows spontaneous fluorescence that is easily observable and therefore is often used as a tracer agent (Li et al., 2011; Song et al., 2016). The concentration of the activated bacterial solution was adjusted to 1.0 × 10^6^ CFU/mL. MH broth was inoculated with the activated bacterial solution at a 1:10 ratio of bacterial solution to medium, and DHA27 at a final concentration of 32, 64 and 128 μg/ml was added, respectively. After incubating at 37°C with shaking at 180 rpm for 6–8 h, the samples were centrifuged at 537 × *g* for 3 min, and the OD_600_ was adjusted to 1.0 by MH broth. DNR was added to the bacterial solution to make the final concentration of DNR 40 μg/ml; the mixtures were incubated at 37°C for 30 min in the dark for 0, 10, 20, and 30 min. Then, 1 ml of bacterial solution from each group at each time point was centrifuged at 537 × *g* for 3 min. The pellet was washed with PBS three times and pipetted and resuspended, and finally the fluorescence intensity of the bacterial solution was measured.

### 2.8 mRNA expressions of 16S rRNA methyltransferases and aminoglycoside-modifying enzymes

Thirteen PA clinical isolates that had antibacterial sensitization of DHA27 in combination with tobramycin were cultured at 37°C with shaking until the OD_600_ value was 0.3–0.8, and the bacteria were collected after centrifugation at 537 *g* for 5 min. RNA extraction and reverse transcription were performed using previously described protocols (Song et al., 2016). Target genes were amplified by real-time fluorescence quantitative PCR using specific primers (Table 1). The 2^−ΔΔCt^ method was used to calculate the expression level of the target gene relative to the reference gene of *16SrRNA* (Livak and Schmittgen, 2001).

**TABLE 1:** Primer sequences.

Genes	Forward (5’ → 3′)	Reverse (5’ → 3′)
**16S-Rmtases**		
*arm-A*	ATG​GAT​AAG​AAT​GAT​GTT​GTT​AAG	TTA​TTT​CTG​AAA​TCC​ACT​AGT​AAT​TA
*rmt-A*	CCT​AGC​GTC​CAT​CCT​TTC​CTC	AGC​GAT​ATC​CAA​CAC​ACG​ATG​G
*rmt-B*	ATG​AAC​ATC​AAC​GAT​GCC​CTC	TTA​TCC​ATT​CTT​TTT​TAT​CAA​GTA​TAT
*rmt-C*	CTC​AAA​CTC​GGC​GGG​CAA​GC	CGA​AGA​AGT​AAC​AGC​CAA​AG
*rmt-D*	ATG​AGC​GAA​CTG​AAG​GAA​AAA​CTG​CT	TCA​TTT​TCG​TTT​CAG​CAC​GTA​AAA​CAG
**AMEs**		
*aac(3)-*I	ACC​TAC​TCC​CAA​CAT​CAG​CC	ATA​TAG​ATC​TCA​CTA​CGC​GC
*aac(3)-*II	ACT​GTG​ATG​GGA​TAC​GCG​TC	CTC​CGT​CAG​CGT​TTC​AGC​TA
*aac(3)-* IV	TCG​ATG​GGC​AGG​TAC​TTC​TC	ACC​GAC​TGG​ACC​TTC​CTT​CT
*aac(6′)-*I*b*	TTG​CGA​TGC​TCT​ATG​AGT​GGC​TA	CTC​GAA​TGC​CTG​GCG​TGT​TT
*aac(6′)-*II	TTC​ATG​TCC​GCG​AGC​ACC​CC	GAC​TCT​TCC​GCC​ATC​GCT​CT
*ant(2″)-*I	GAG​CGA​AAT​CTG​CCG​CTC​TGG	CTG​TTA​CAA​CGG​ACT​GGC​CGC
*ant(2″)-*I*a*	ATG​GAC​ACA​ACG​CAG​GTC​GC	TTA​GGC​CGC​ATA​TCG​CGA​CC
*ant(3″)-*I	TGA​TTT​GCT​GGT​TAC​GGT​GAC	CGC​TAT​GTT​CTC​TTG​CTT​TTG
*aph(3′)-*I*a*	CGA​GCA​TCA​AAT​GAA​ACT​GC	GCG​TTG​CCA​ATG​ATG​TTA​CAG
**16S rRNA**	GAG​AGA​AGG​TGG​GGA​TGA​CGT	AGG​CCC​GGG​AAC​GTA​TTC​AC

16S-Rmtases, 16S rRNA, methyltransferase; AMEs, Aminoglycoside modification enzymes.

### 2.9 Analysis of effects of DHA27 on tobramycin metabolites

The concentration of the activated bacterial solution was adjusted to 1.0 × 10^6^ CFU/ml. According to the optimal concentration of DHA27 in combination with TOB to exert the greatest antibacterial sensitization effect, the groups were established as follows: broth group, DMSO group, 1/16 MIC TOB (64 μg/ml) group and 1/16 MIC TOB (64 μg/ml) + 1/16 MIC DH27 (64 μg/ml) group. The bacterial solution was, incubated at 37°C with constant-temperature shaking at 180 rpm for 6–8 h and centrifuged at 537 × *g* for 3 min. The supernatant was discarded; the pellet was washed with PBS and resuspended with 1 ml of triple-distilled water. The solution was centrifuged at 4,480 × *g* for 10 min at 4°C; the supernatant was discarded, and the bacterial pellet washed with and resuspended in triple-distilled water. Bacteria were lysed by ultrasonication (ultrasonic power 300–350 W, working 9.9 s, intermittent 9.9 s; 152 cycles) in an ice bath.

Tobramycin metabolites were detected by high-resolution UPLC-Q/Orbitrap MS in the supernatant of the disrupted bacterial liquid after sonication. Separations were performed on a Hypersil GOLD C18 column (150 × 2.1 mm, 1.9 μm, Thermo Fisher Scientific, Bellefonte, PA, USA). The mobile phase consisted of 0.1% formic acid solution (A) and methanol (B) with a flow rate of 0.3 ml/min 0–1.5 min, 30% B; 1.5–9 min, linear from 30% to 80% B, and kept for 7.5 min at 80%,16.5–18 min, linear from 80% to 30% B, and kept for 2 min at 30%. The temperatures of the column oven and autosampler were set at 40°C and 8°C, respectively. Injection volume was 3 μL. The following conditions were used for positive ESI mode: spray voltage, 3.5 kV; capillary temperature, 350°C; sheath gas, 35.00 units; auxiliary gas, 15.00 units; aux gas heater temperature, 350°C. Data was acquired using Full Scan-ddMS2 scan mode.

### 2.10 Statistical analysis

All data were expressed as mean ± standard deviation. Statistical analysis was performed using one-way ANOVA, two-tailed unpaired Student’s t-test and Pearson’s correlation analysis in GraphPad Prism 8.0.1 software. A *p* value of 0.05 was considered significant, and a *p* value of 0.01 was considered highly significant.

## 3 Results

### 3.1 *DHA27* had no direct antibacterial effect but it can increase the antibacterial activity of aminoglycosides in *Pseudomonas aeruginosa*

The MIC of DHA27 was >1,024 μg/ml for PA standard strain, indicating that DHA27 had no direct antibacterial effect on PA. The combination of DHA27 and tobramycin had a FICI of 0.28 and the FICI <0.5 we considered that the combination of the two drugs had a synergistic antibacterial effect. DHA27 combined with TOB had an antibacterial sensitization effect on PA ATCC27853 (Tables 2 and 3). Therefore, gentamicin, TOB, and amikacin were selected for further experiments performed in combination with DHA27 to observe their antibacterial sensitization effect on 20 clinical isolates of PA.

**TABLE 2:** Minimum inhibitory concentrations (MICs) of DHA27 and different aminoglycosides for *Pseudomonas aeruginosa* (PA) ATCC27853 and 20 clinical isolates of PA.

PA	GM	TOB	AMI	DHA27
ATCC27853	<0.5	1	2	>1,024
clinical isolates	128–1,024	256–1,024	16–1,024	>1,024

GM, gentamicin; TOB, tobramycin; AMI, amikacin. The MIC, range indicates the interval of the MICs, of antimicrobial drugs for different clinical isolates of PA.

**TABLE 3:** Fractional inhibitory concentration index (FICI) values for DHA27 in combination with aminoglycosides against *Pseudomonas aeruginosa* (PA) ATCC27853 and 20 clinical isolates of PA.

Strain/combination	Bacterial resistance rate (%)^a^	FICI^b^	
PA ATCC27853			
¼ MIC TOB+32 μg/ml DHA27		0.28
PA clinical isolates			
GM + DHA27	100.0	0.17 ± 0.17 (16/20)	
TOB + DHA27	100.0	0.32 ± 0.15 (13/20)	
AMI + DHA27	35.0	0.41 ± 0.12 (9/20)	

^a^ Bacterial resistance rate refers to the resistance rate of 20 PA, clinical isolates to gentamicin (GM), tobramycin (TOB) and amikacin (AMI).

^b^ FICI, data is expressed as mean ± standard deviation; the values in brackets indicate the ratio of the number of bacteria with antibacterial sensitization to the total number of bacteria.

The results showed that the 20 PA clinical isolates resistant rates to gentamicin, tobramycin and amikacin was 100%, 100% and 35%, respectively (Table 3). Because AGs are the most commonly used drugs for the clinical treatment of PA infection, we focused on the resistance of PA to TOB, a representative aminoglycoside antibiotic. The results showed that the MIC of TOB against 20 clinical isolates of PA was ≥256 μg/ml. According to the 2020 CLSI guidelines for resistance of PA to TOB at an MIC ≥16 μg/ml, the 20 clinical isolates of PA were highly resistant to TOB. The MIC of DHA27 to all 20 clinical isolates of PA was ≥1,024 μg/ml. The MICs of DHA27 were >1,024 μg/ml for PA clinical isolates, indicating that DHA27 had no direct antibacterial effect against the 20 clinical isolates of PA; however, DHA27 significantly enhanced the antibacterial activity of aminoglycoside antibiotics against most of the clinical isolates of PA, the FICIs <0.5 (Table 3).

### 3.2 DHA27 combined with tobramycin can reduce the bacterial loads and proinflammatory factor levels in sublethal sepsis mouse model

**FIGURE 1:**
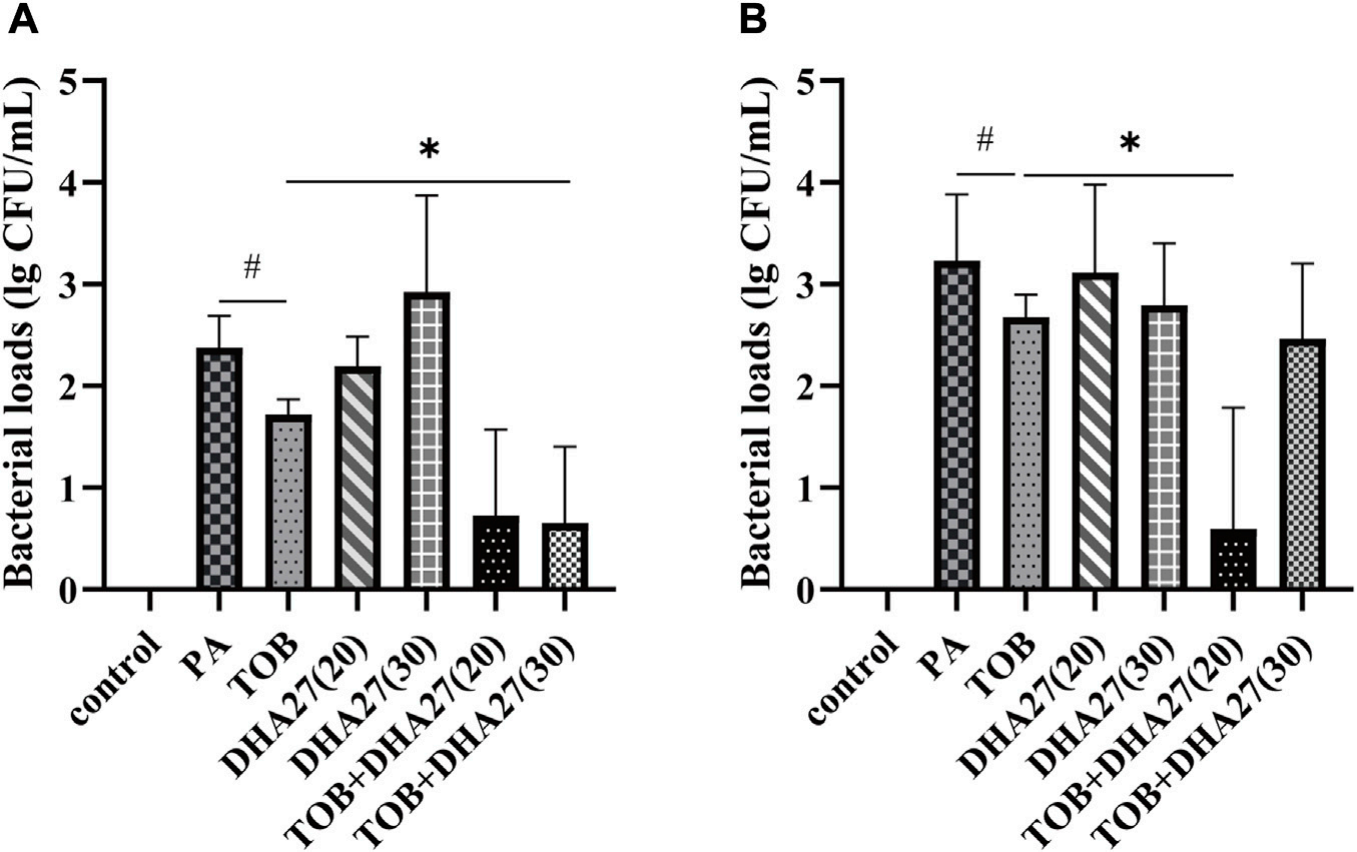
Bacterial load in the spleen and lungs of sepsis model mice **(A)** spleen **(B)** lungs (mean ± standard deviation; *n* = 4); DHA27 (20): DHA27 20 mg/kg/d; DHA27 (30): DHA27 30 mg/kg/d; *p < 0.05 vs. tobramycin (TOB) group.

Because DHA27 combined with TOB had an antibacterial sensitization effect on PA *in vitro*, we investigated the pharmacodynamics of the drug combination *in vivo*. The results showed that the bacterial load in the spleen homogenate was higher in the PA group than in the control group. The bacterial load was lower in the TOB group than in the PA group, but there was no statistically significant difference (*p* > 0.05). However, the bacterial load in the spleen was lower in the DHA27 + TOB group than in the TOB group. In particular, TOB combined with 30 mg/kg/d DHA27 significantly reduced the bacterial load in the spleen (*p* < 0.05; Figure 1A).

The bacterial load of the lung homogenate was higher in the PA group than in the control group. When TOB was added alone, the bacterial loads were lower in the TOB group than in the PA group, but there was no statistically significant difference (*p* > 0.05). However, the bacterial load in the lungs was lower in the DHA27 + TOB group than in the TOB group. In particular, TOB combined with 20 mg/kg/d DHA27 significantly reduced the bacterial load in spleen (*p* < 0.05; Figure 1B).

**FIGURE 2:**
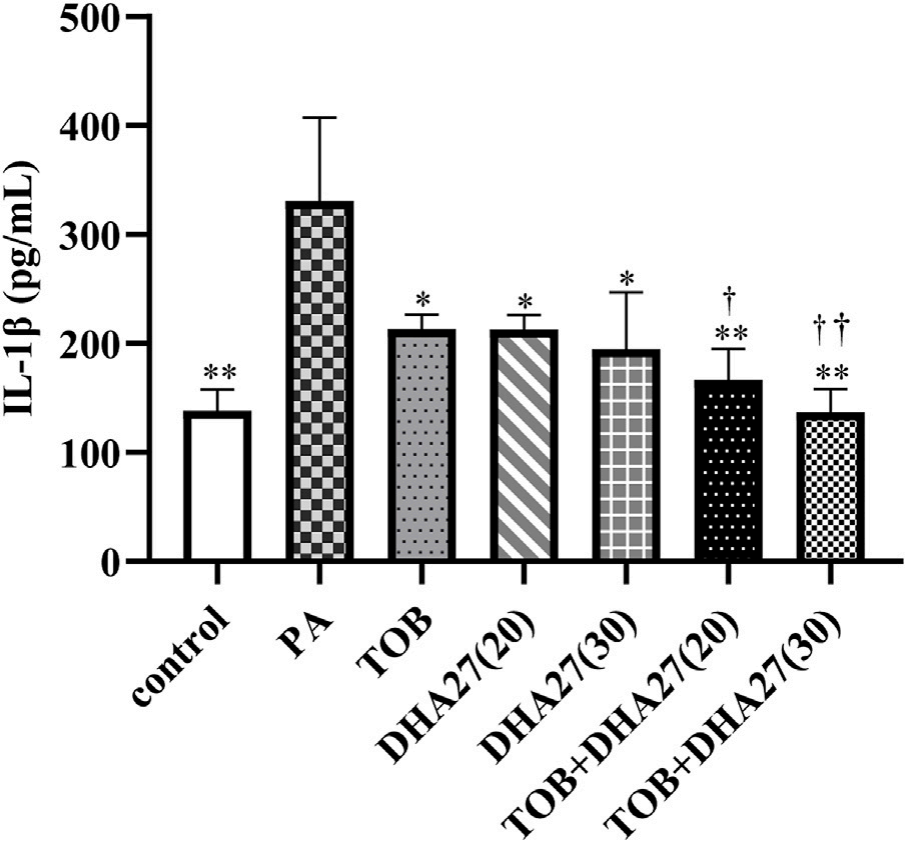
Effects of DHA27 combined with tobramycin (TOB) on the levels of interleukin (IL)-1β in the spleen of sepsis model mice (mean ± standard deviation; *n* = 4); DHA27 (20): DHA27 20 mg/kg/d; DHA27 (30): DHA27 30 mg/kg/d; *p < 0.05, **p < 0.01 vs. PA group; ^†^ *p* < 0.05, ^††^ *p* < 0.01 vs. TOB group.

The IL-1β level in the spleen was significantly higher in the PA group than in the control group (*p* < 0.01). The level of IL-1β in the spleen in the TOB group and the two DHA27 groups was significantly lower than that in the PA group (*p* < 0.05). The DHA27 + TOB treatments significantly reduced the level of IL-1β in comparison with the TOB treatment (*p* < 0.01; Figure 2).

**FIGURE 3:**
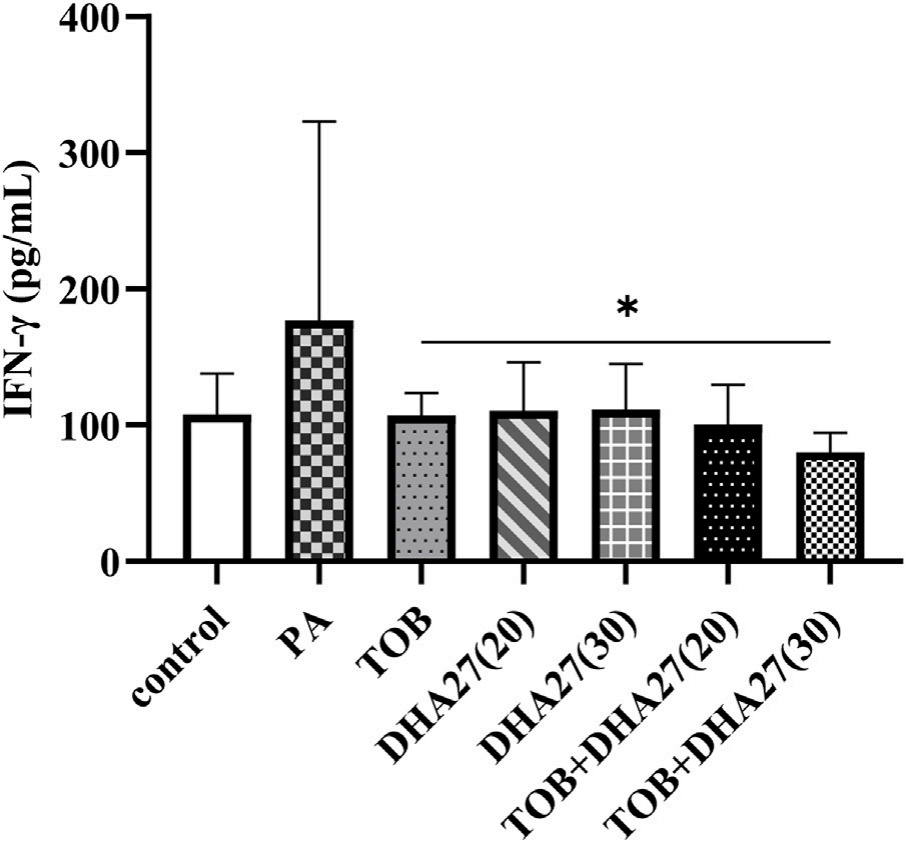
Effects of DHA27 combined with tobramycin (TOB) on the levels of interferon (IFN)-γ in the lungs of sepsis model mice (mean ± standard deviation; *n* = 4); DHA27 (20): DHA27 20 mg/kg/d; DHA27 (30): DHA27 30 mg/kg/d; *p < 0.05 vs. TOB group.

The IFN-γ level in the lungs was slightly higher in the PA group than in the control group, but there was no statistically significant difference (*p* > 0.05). The IFN-γ level in the TOB group was lower than that in the PA group, but the difference was not statistically significant (*p* > 0.05). However, TOB combined with 30 mg/kg/d DHA27 significantly reduced the level of IFN-γ in the lungs (*p* < 0.05; Figure 3).

### 3.3 DHA27 does not exacerbate the damage induced by tobramycin to *Pseudomonas aeruginosa morphology*

**FIGURE 4:**
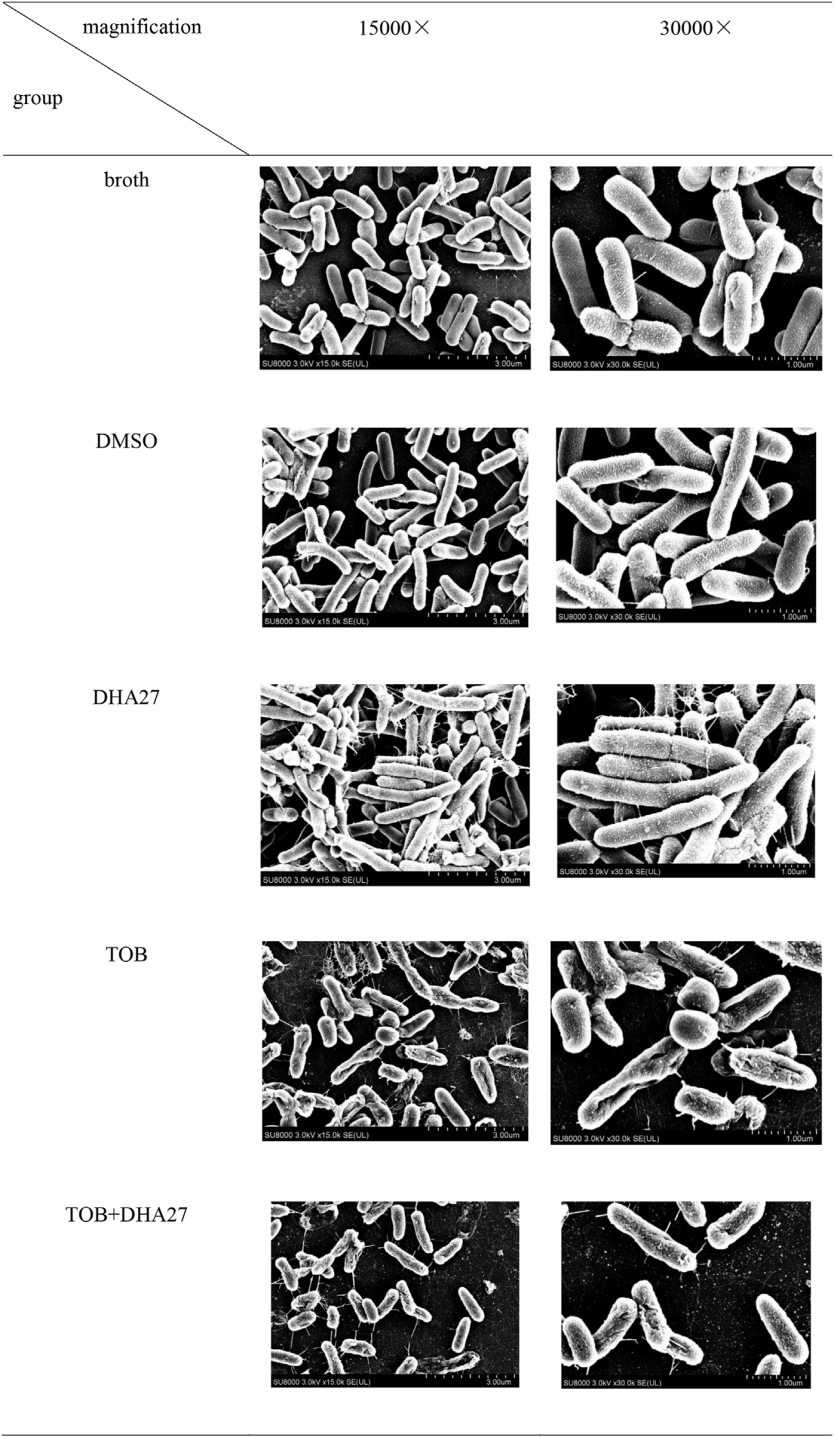
Effects of DHA27 in combination with tobramycin (TOB) on the morphological structure of PA16 as observed using scanning electron microscopy.

Next, we investigated whether DHA27 plays a role by directly damaging bacterial morphology. SEM showed that bacteria in the broth group had a smooth surface without burrs. Compared with the broth group, the DMSO group bacteria were intact in shape and structure. This observation indicated that DMSO did not directly damage the morphological structure of PA. Compared with the broth group, the bacterial morphology in the DHA27 group was still relatively unchanged, and the structure was not significantly damaged, indicating that DHA27 did not directly destroy the morphology and structure of PA. Compared with the broth group, the surface of bacteria in the TOB group was rough. It showed a certain reduction in the number and sizes of bacteria in the TOB group were also lower than that in the broth group. The concave structure on the surface of the bacterial cells was damaged to a certain extent. Thus, TOB had an antibacterial effect on PA, and it damaged its morphological structure. The number of bacteria in the DHA27 + TOB group was slightly lower than that in the TOB group. Regarding bacterial cell morphology in the DHA27 + TOB group, the bacterial surface showed depressions and damage to a certain extent. However, compared with the TOB group, the degree of damage to bacterial morphology did not increase significantly. Thus, in the DHA27 + TOB group, DHA27 did not induce further direct damage to the morphology of PA in comparison with the TOB group (Figure 4).

### 3.4 DHA27 does not significantly increase daunorubicin accumulation in *Pseudomonas aeruginosa*

Drug accumulation in bacterial cells is an important mechanism of bacterial drug resistance. The results showed that in 13 PA clinical isolates with antimicrobial sensitization to DHA27 in combination with tobramycin, DHA27 at different concentrations increased the fluorescence intensity at certain time points (Supplementary Table S1). FICI <0.5 indicated that the combination of drugs had a synergistic antibacterial effect; the lower the FICI level, the better the synergistic effect of the two drugs. However, further correlation analysis was conducted between the FICI value and the increase in fluorescence intensity in bacteria after DHA27 was added at 30 min. The results showed that the DHA27-induced increase in fluorescence intensity in bacteria and FICI were not significantly correlated (Table 4). Therefore, the effect of DHA27 on antimicrobial sensitization was not related to the increase in drug accumulation in bacteria.

**TABLE 4:** Correlation analysis between fractional inhibitory concentration index (FICI) values and enhanced daunorubicin (DNR) accumulation in 13 clinical isolates of *Pseudomonas aeruginosa*.

		FICI					*R* ^*2*^	*P*
		<0.13	0.13–0.19	0.19–0.25	0.25–0.31	0.31–0.38		
		(n = 2)	(n = 6)	(n = 2)	(n = 2)	(n = 1)		
Increased accumulation of DNR (%)	DHA27 (32)	0 (0.0)	2 (33.3)	1 (50.0)	1 (50.0)	0 (0.0)	0.0055	0.9057
	DHA27 (64)	0 (0.0)	2 (33.3)	0 (0.0)	2 (100.0)	0 (0.0)	0.048	0.7233
	DHA27 (128)	0 (0.0)	2 (33.3)	0 (0.0)	2 (100.0)	0 (0.0)	0.048	0.7233

### 3.5 DHA27 does not inhibit the mRNA expression of 16S rRNA methyltransferases

After several representative 16S rRNA methyltransferase (16S-Rmtase) to study the effect of DHA27 in combination with TOB on the mRNA expression of 16S-Rmtases were selected (Supplementary Table S2), the correlation between the mRNA expression of 16S-Rmtases and FICI in 13 clinical isolates of PA with antimicrobial sensitization was analyzed. The FICI values were divided into five groups according to the synergistic antibacterial effect of DHA27 combined with TOB, and the difference in 16S-Rmtase gene expression was compared among the different FICI groups. The results showed that there was no significant correlation between the mRNA expression of 16S-Rmtases and FICI value (Table 5), indicating that DHA27 did not inhibit the mRNA expression of 16S-Rmtases.

**TABLE 5:** Correlation analysis between fractional inhibitory concentration index (FICI) values and 16S rRNA methyltransferase (16S-Rmtase) mRNA expression in 13 clinical isolates of *Pseudomonas aeruginosa*.

Subtype		FICI					*R* ^*2*^	*P*
		<0.13 (n = 2)	0.13–0.19 (n = 6)	0.19–0.25 (n = 2)	0.25–0.31 (n = 2)	0.31–0.38 (n = 1)		
Percentage reduction of 16S-Rmtase expression (%)	*armA*	2 (100.0)	2 (33.3)	0 (0.0)	1 (50.0)	1 (100.0)	0.0071	0.8930
	*rmtA*	2 (100.0)	3 (50.0)	0 (0.0)	1 (50.0)	1 (100.0)	0.0006	0.9690
	*rmtB*	1 (50.0)	1 (16.7)	1 (50.0)	1 (50.0)	0 (0.0)	0.2199	0.4256
	*rmtC*	1 (50.0)	2 (33.3)	1 (50.0)	0 (0.0)	0 (0.0)	0.6947	0.0795
	*rmtD*	1 (50.0)	3 (50.0)	0 (0.0)	1 (50.0)	1 (100.0)	0.2196	0.4260

### 3.6 DHA27 combined with tobramycin inhibits the mRNA expression of aminoglycoside-modifying enzymes

Since antibacterial sensitization effect of DHA27 was not mainly achieved by directly damaging the morphological structure of bacteria, increasing the accumulation of antibacterial drugs in bacterial cells, and inhibiting the mRNA expression of 16S-Rmtases, and enzymatic modification of aminoglycosides is the most important mechanism of aminoglycoside antibiotic resistance (Ramirez and Tolmasky, 2010; Wachino et al., 2020), therefore, it was experimentally investigated whether DHA27 inhibited the mRNA expression of AMEs to exert an antibacterial sensitization effect.

After the correlation between AME mRNA expression level and antibacterial sensitization activity of DHA27 were analyzed, the FICI values were divided into five groups according to the synergistic antibacterial effect of combination drugs and compared the differences in AME gene expression among the FICI groups. The results showed that there was a statistically significant difference among the groups in the percent reduction in mRNA expression of *aac(3)-Ⅱ* (*p* < 0.05). As FICI increased, the synergistic antibacterial effect decreased, and the ability to lower the expression level of *aac(3)*-II decreased, too (Table 6).

**TABLE 6:** Correlation analysis between fractional inhibitory concentration index (FICI) values and mRNA expression of aminoglycoside-modifying enzymes (AMEs) in 13 clinical isolates of *Pseudomonas aeruginosa*.

Subtype		FICI					*R* ^*2*^	*P*
		<0.13 (*n* = 2)	0.13–0.19 (n = 6)	0.19–0.25 (n = 2)	0.25–0.31 (n = 2)	0.31–0.38 (n = 1)		
Percentage reduction in AME mRNA expression (%)	*aac(3)-Ⅰ*	2 (100.0)	2 (33.3)	0 (0.0)	1 (50.0)	1 (100.0)	0.0071	0.8930
	*aac(3)-Ⅱ*	2 (100.0)	2 (33.3)	1 (50.0)	0 (0.0)	0 (0.0)	0.7784	**0.0476**
	*aac(3)-Ⅳ*	2 (100.0)	4 (66.7)	0 (0.0)	1 (50.0)	0 (0.0)	0.6207	0.1135
	*aac(6′)-Ⅰb*	2 (100.0)	3 (50.0)	0 (0.0)	2 (100.0)	1 (100.0)	0.0354	0.7619
	*aac(6′)-Ⅱ*	2 (100.0)	4 (66.7)	1 (50.0)	1 (50.0)	1 (100.0)	0.0055	0.9057
	*ant(2″)-Ⅰa*	2 (100.0)	3 (50.0)	0 (0.0)	1 (50.0)	1 (100.0)	0.0006	0.9690
	*ant(2″)-Ⅰ*	1 (50.0)	3 (50.0)	1 (50.0)	1 (50.0)	1 (100.0)	0.5322	0.1618
	*ant(3″)-Ⅰ*	0 (0.0)	3 (50.0)	2 (100.0)	1 (50.0)	0 (0.0)	0.0006	0.9690
	*aph(3′)-Ⅰa*	1 (50.0)	2 (33.3)	0 (0.0)	1 (50.0)	0 (0.0)	0.2819	0.3573

R^2^ stands for the squared value of the correlation coefficient, *P* stands for regression coefficient.

### 3.7 DHA27 decreases tobramycin metabolites in bacteria treated with tobramycin

**FIGURE 5:**
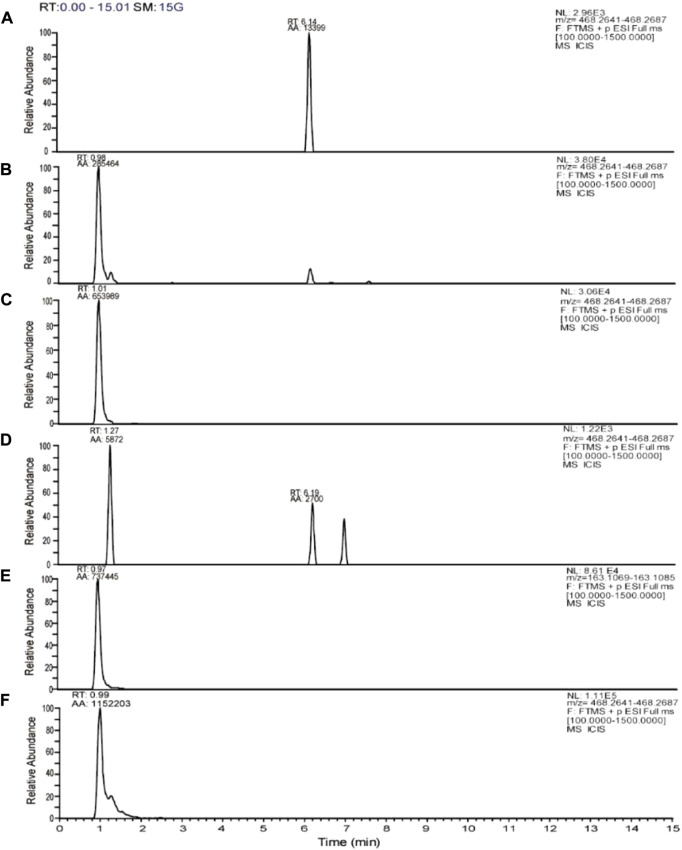
Representative extracted ion chromatograms (EICs) of tobramycin obtained from **(A)** ATCC27853 broth group **(B)** ATCC27853 TOB group **(C)** ATCC27853 DHA27 + TOB group **(D)** PA16 broth group **(E)** PA16 TOB group **(F)** PA16 DHA27 + TOB group.

Tobramycin chromatographic peak was not detected in the PA standard strain ATCC27853 standard strain broth group (Figure 5A), but tobramycin peaks were observed in standard strain TOB group and DHA27 + TOB group at m/z 468.26. Moreover, the peak area of tobramycin increased after DHA27 was added (Figures 5B,C), indicating that the modification of tobramycin by the PA standard strain decreased after DHA27 was added. Tobramycin chromatographic peak was not detected in the clinical isolate PA16 broth group (Figure 5D), and the TOB peaks at m/z 163.11 and m/z 468.26 were observed in PA16, respectively. However, the peak area of tobramycin increased after the addition of DHA27 (Figures 5E,F), indicating that the modification of tobramycin by PA clinical isolate decreased after the addition of DHA27.

**FIGURE 6:**
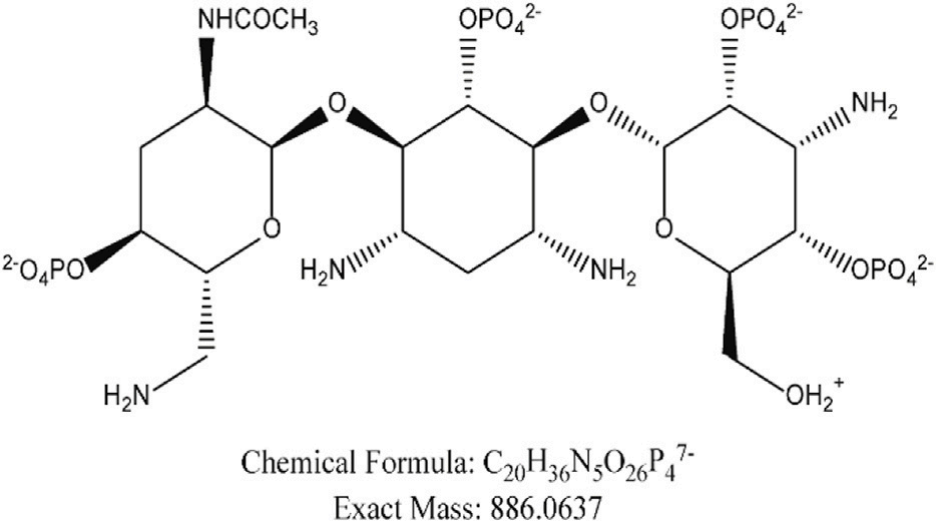
Chemical structure modified by tobramycin derivatization.

**FIGURE 7:**
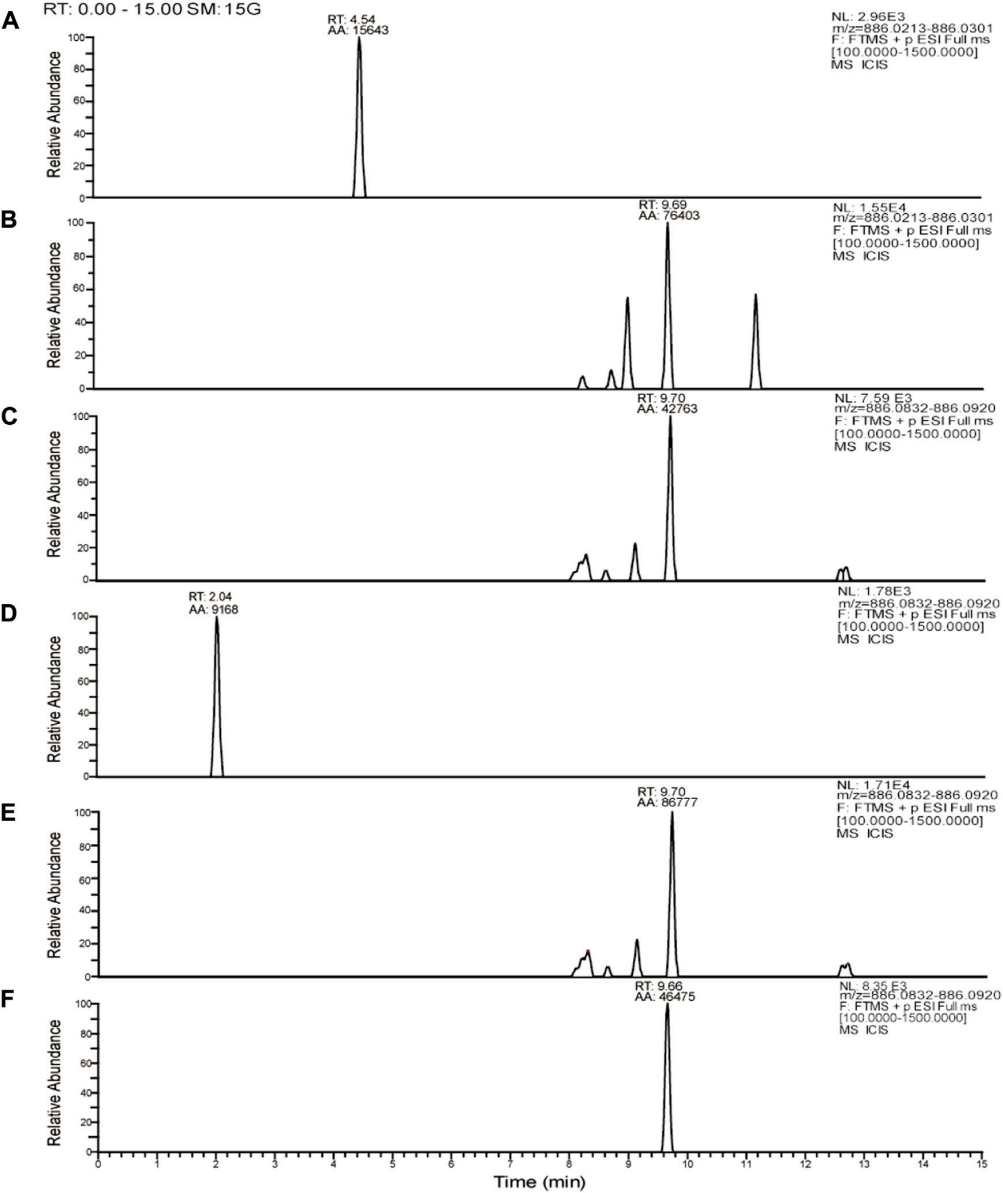
Representative extracted ion chromatograms (EICs) of tobramycin derivatization obtained from**(A)** ATCC27853 broth group **(B)** ATCC27853 TOB group **(C)** ATCC27853 DHA27 + TOB group **(D)** PA16 broth group **(E)** PA16 TOB group **(F)** PA16 DHA27 + TOB group.

We compared our results with the acetylation products of tobramycin in the literature, and we found that the peak in the extracted ion flow diagram RT:9.69 was the acetylation product with the chemical formula C_20_H_36_N_5_O_26_P_4_^7-^ (m/z 886.06). (Figure 6) The tobramycin derivatization chromatographic peak was not seen in the broth group of the standard strain (Figure 7A), Further addition of DHA27 compared with TOB group, the peak area of derivatized tobramycin in DHA27 + TOB group decreased (Figures 7B,C). The results showed that the peak area of acetylation products in TOB + DHA27 group was reduced compared with that in TOB group in the PA standard strain. No tobramycin peak was observed in the clinical isolate PA16 (Figure 7D). However, derivatized tobramycin peaks were found in both TOB group and DHA27 + TOB group of PA16. But the peak area of derivatized tobramycin decreased after the addition of DHA27 (Figures 7E,F), indicating that the addition of DHA27 could reduce the modification of tobramycin by PA clinical isolate.

## 4 Discussion

To our knowledge, this is the first study to demonstrate that DHA27 in combination with AGs has a certain antimicrobial sensitization effect on PA. In this study, the AG resistance of 20 clinical isolates of PA from Zunyi, Guizhou was determined. Most of the 20 clinical isolates of PA were resistant to all tested AGs, which signify the gravity of drug resistance of PA; thus, it is imminent to study novel strategies for the use of AGs.

PA is a common Gram-negative bacterium that causes nosocomial and chronic infections. The drug resistance of PA is an important reason for its high clinical isolation rate and refractoriness to treatment. The main drug resistance mechanisms of PA include enzymatic modification of antibiotic drugs, activation of a drug efflux pump system, changes in outer membrane permeability, and gene mutation (Breidenstein et al., 2011). AGs, quinolones, and β-lactams are common clinical antibiotics currently used to treat infections caused by PA (Vardakas et al., 2013; Goli et al., 2016; Akhi et al., 2017). With the increase in bacterial resistance to β-lactams and fluoroquinolone antibiotics in clinical use, AGs have gradually begun to be considered as the first choice for the treatment of life-threatening, serious infections caused by PA (Odumosu et al., 2015; Belaynehe et al., 2017).

To overcome the challenge of bacterial resistance, in addition to developing new antibacterial drugs, structural modifications of existing antibiotics to restore their antibacterial activity is a noteworthy approach. AGs exert an antibacterial effect by binding to the A site of the 16S rRNA of the 30S small subunit of the bacterial ribosome and interfering with protein translation (Carter et al., 2000; Magnet and Blanchard, 2005). Plazomicin (PLA) is a novel aminoglycoside antibiotic generated by a synthetic modification of N1 and N6 positions of sisomicin (Aggen et al., 2010). In 2018, it was approved by the FDA for clinical application in the treatment of complicated urinary tract infection and pyelonephritis (Golkar et al., 2021; "Plazomicin (Zemdri) - a new aminoglycoside antibiotic," 2018). Owing to its structural modifications, PLA blocks the AME binding site, which enhances its antibacterial activity. However, in drug research and development, it is difficult and time consuming to restructure existing antibiotics to identify novel targets for drug action, which is a limiting factor considering the rapid emergence of bacterial drug resistance. Indirect antibacterial strategy refers to the combination of compounds with no antibacterial activity and antibiotics to change and modify the bacterial phenotype to exert an antibacterial sensitization effect; such compounds are called antibacterial sensitizers (Worthington and Melander, 2013; Wright, 2016; Tyers and Wright, 2019). In recent years, antibacterial sensitizers have gradually become the new choice to manage bacterial drug resistance owing to the easy availability of raw materials and the absence of selection pressure on bacteria.

Our previous study found that the artemisinin derivative artesunate combined with β-lactam antibiotics has an antibacterial sensitization effect on *Escherichia coli* (Li et al., 2008) and Methicillin resistant *Staphylococcus aureus* (Jiang et al., 2011). The main mechanism underlying the effect of artesunate in enhancing the sensitivity of *E. coli* to β-lactam antibiotics is to inhibit the expression of the active efflux pump transporter AcrB in *E. coli*. Using this mechanism as the target, computer-aided drug design was used to structurally modify artemisinin and its derivatives. Among the new synthetic artemisinin derivatives, DHA7 had the strongest antibacterial sensitization effect, and its mechanism of action may also be closely related to the expression of *AcrB* mRNA (Wu et al., 2013).

However, owing to the poor antibacterial and sensitizing activity of artesunate and DHA7 in clinical isolates, the branched chain structure of artemisinin was further modified. It was found that the combination of artemisinin derivative DHA27 with β-lactam antibiotics has a certain antibacterial and sensitizing activity against an *E. coli* standard strain and clinical isolates of *E. coli*, but its combination with non-β-lactam antibiotics had poor activity (Song et al., 2016). In the present study, the antibacterial sensitization effect and molecular mechanisms of DHA27 combined with aminoglycosides were studied, to investigate the compatibility of DHA27 with other antibacterial drugs and explore its potential for a superior antibacterial sensitization effect.

The present findings suggest that DHA27 combined with TOB had an antibacterial sensitization effect on PA. FICI measurement *in vitro* and the protective effect of DHA27 in combination with TOB on sepsis model mice *in vivo* both indicated that DHA27 had good antibacterial sensitization effects.

The research and development of antibacterial sensitizers mainly depends on the mechanism of antibiotic resistance. At present, the mechanisms of antibiotic resistance is divided into the following categories (Munita and Arias, 2016; Yelin and Kishony, 2018): 1) the action of antibiotic-modifying enzymes leading to the inactivation of antibiotics; 2) increase in drug efflux, which reduces antibiotics in the bacterial cells; 3) direct alteration of the antibiotic or mutation of the action target of the antibiotic; 4) changes in the process of drug metabolism, which makes the antibiotic inactive. In the present study, scanning electron microscopy observations showed that DHA27 did no direct damage to bacterial morphology.

Drug accumulation plays an important role in drug resistance of PA, in which the active efflux pump comprising the MexXY protein and outer membrane component OprM plays an important role in the adaptive resistance of PA to AGs (Aires et al., 1999; Mine et al., 1999; Hocquet et al., 2003). Phenylalanyl-arginyl-β-naphthylamide (PaβN), a broad-spectrum efflux pump inhibitor, inhibits MexXY-OprM and other efflux pumps in PA (Opperman and Nguyen, 2015). Owing to the cationic groups inherent in the structure of PaβN, it accumulates in the kidney tissue for a long time and causes severe renal toxicity, and a series of structural modifications to PaβN have not led to substantial progress (Opperman and Nguyen, 2015). Therefore, currently, there are no suitable inhibitors of active efflux pumps. We found that FICI, which signifies synergistic antibacterial effects, was not associated with increased accumulation of daunorubicin in bacteria, suggesting that the antimicrobial sensitization effect of DHA27 is not exerted by increasing AG accumulation in PA.

Acquired 16S rRNA methyltransferase (16S-Rmtase) is one of the main mechanisms of aminoglycoside antibiotic resistance in bacteria. The enzyme uses S-adenosine methionine (SAM) as a cofactor to add methyl groups to specific residues of 16S rRNA (Wang et al., 2022). AGs have a lower affinity for methylated 16S rRNA than for non-methylated 16S rRNA, which makes the bacteria resistant to a broad spectrum of AGs (Wachino et al., 2020). 16S-Rmtases are divided into two types according to their methylation sites: those that methylate the N7 position of nucleotide G1405 and those that methylate the N1 position of nucleotide A1408. Subsequently, a total of nine N7-G1405 16S-RMTases were identified as ArmA, RmtA, RmtB, RmtC, RmtD, RmtE, RmtF, RmtG, and RmtH. Previous studies have reported that 16S-Rmtases such as *arm-A* in *K. pneumoniae* and *rmt-A* in PA mediate aminoglycoside resistance (Galimand et al., 2003; Yokoyama et al., 2003). At present, there is no report on the effect of acquired 16S-Rmtases affect clinical diagnosis and treatment of patients using AGs, the reason probably is AGs are currently not used as first-line drugs for Gram-negative bacterial infections and the detection rate of 16S-Rmtases is low in developed countries (Doi et al., 2016).

AGs act on bacterial ribosomes to inhibit bacterial protein synthesis. In 1997, Japanese scientists discovered that a 16S-Rmtase, subsequently named *rmt-*A, mediates the high resistance of PA to AGs (Yokoyama et al., 2003). Since then, several exogenous 16S-Rmtases have been found successively, and these enzymes have been found to cause extensive and high level of resistance of pathogenic Gram-negative bacteria to AGs (Galimand et al., 2003). The mechanism of action of 16S-Rmtases is the addition of a CH_3_ group to specific nucleotide residues at the 16S rRNA A site, resulting in a decreased affinity of AGs to the methylated 16S rRNA, which confers resistance. In the present study, DHA27 combined with TOB did not significantly inhibit the mRNA expression of 16S-Rmtases, indicating that exerts the antibacterial sensitization effect of DHA27 is not exerted through inhibition of the mRNA expression of 16S-Rmtases.

The main resistance mechanism of PA to AGs is the enzymatic modification of AGs. After modification, the amino or hydroxyl groups of AGs cannot remain tightly bound to the ribosome and cannot enter the next stage of antibacterial action, enabling bacteria to survive in the presence of AG antibiotics (Llano-Sotelo et al., 2002). Some studies suggest that the antimicrobial resistance genes in PA can be transmitted between bacteria, and the transfer of drug resistance genes (Hasani et al., 2016), especially in the hospital environment, further aggravates the infection (Odumosu et al., 2015). Genes encoding AMEs are among the drug resistance genes transferred between PA (Belaynehe et al., 2017). AMEs are divided into three categories according to the subunits of aminoglycoside antibiotics that are modified: acetyltransferases (AACs), phosphotransferases, and nucleotidyltransferases (Ramirez and Tolmasky, 2010). Movable genetic elements such as integrons, transposons and plasmids facilitate the rapid spread of these drug resistance genes among bacteria (Wright, 1999). Therefore, it is important to investigate whether inhibition of AME function or mRNA expression is related to improved bacterial resistance.

In this study, focusing on various representative subtypes of AMEs, our results showed that TOB induced high the mRNA expression of AMEs, whereas DHA27 combined with TOB inhibited the high expression. The correlation between FICI and the rate of AME mRNA expression inhibition demonstrated that the antibacterial sensitization effect of DHA27 was most closely related to the inhibition of the mRNA expression of acetyltransferase *aac(3)-Ⅱ*.

AACs use acetyl-CoA as a donor of acetyl groups and act at different sites of AGs to modify most AGs used in clinical practice. If DHA27 increased the inhibition of AAC expression, a reduction in acetylation products would be observed. The results of mass spectrometry showed that DHA27 reduced the acetylation products in the bacterial supernatant, further indicating that DHA27 indeed inhibited AAC mRNA expression. Whether DHA27 inhibits the function of AACs remains to be further studied.

As we know, AMEs is the major reason to induce PA resistance to AGs, so a drug that can inhibit the activity or expression of the AMEs can act as an antimicrobial sensitizer. Even if this drug has no direct antibacterial effect, it can also play a good synergistic effect because it affects the function and expression of drug-resistant enzymes. Our results showed DHA27 inhibits the mRNA expression of AMEs, so TOB is not destroyed by resistant enzymes in the bacteria; high concentration of TOB can exert its antibacterial effect against PA.

## 5 Conclusion

In summary, DHA27 has no direct antibacterial effect; however, in combination with AGs, DHA27 exerts an antibacterial sensitization effect on PA, which is closely related to the inhibition of *aac(3)-*II mRNA expression in PA.

## Data Availability

The original contributions presented in the study are included in the article/Supplementary Materials, further inquiries can be directed to the corresponding authors.
