# Supplementary material for: Artemisinin derivative DHA27 enhances the antibacterial effect of aminoglycosides against Pseudomonas aeruginosa by inhibiting mRNA expression of aminoglycoside-modifying enzymes
Source: Front Pharmacol. 2022 Oct 24;13:970400. doi: 10.3389/fphar.2022.970400 (PMC9637796; doi:10.3389/fphar.2022.970400)
Supplement: Supplementary file 2 [file DataSheet1.pdf]

Supplementary Materials

Supplementary Table 1

Effects of DHA27 (32, 64, and 128 μg/mL) on daunorubicin (DNR) accumulation in clinical isolates of *Pseudomonas aeruginosa* (PA)

isolates name	FICI	Incubation time after adding daunorubicin								
		10 min			20 min			30 min		
		DHA27(32)	DHA27(64)	DHA27(128)	DHA27(32)	DHA27(64)	DHA27(128)	DHA27(32)	DHA27(64)	DHA27(128)
PA05	<0.13	↓↓	↓↓	↓↓	↓↓	↓↓	↓↓	↓↓	↓↓	↓↓
PA16	<0.13	↓	↓	↓	↑↑	—	↓↓	↓↓	—	—
PA01	<0.19	—	↓	↓	↑↑	—	—	↑↑	—	—
PA06	<0.19	—	—	↓↓	—	↓	↓↓	—	—	↓↓
PA07	<0.19	—	—	↓↓	↓↓	↓↓	↓↓	—	↑	↓
PA08	<0.19	↓	↓↓	↓↓	—	↓	↓↓	↓↓	—	↓↓
PA13	<0.19	↑↑	↑↑	↑↑	↑↑	—	↓↓	↑↑	↑↑	↑↑
PA17	<0.19	—	↑↑	—	—	↑↑	↑↑	↓	—	↑↑
PA04	<0.25	↑↑	↑↑	↑↑	—	↓↓	↑↑	↑↑	↓↓	↓↓
PA09	<0.25	—	↓	↓↓	—	↓↓	↓↓	↓↓	↓↓	↓↓
PA02	<0.31	↑↑	—	—	↓↓	↑↑	↓↓	↑↑	↑↑	↑↑

PA19	<0.31	↑↑	↑↑	↑↑	↑↑	↑↑	↑↑	↓	↑↑	↑
PA20	<0.38	—	—	—	↑↑	—	↓↓	↓↓	—	↓↓

FICI, fractional inhibitory concentration index. ↑↑, the increase in the DHA27 group compared with the DNR group was statistically different; ↑, Compared with the DNR group, DHA27 group showed an upward trend but no statistically significant difference; —, DHA27 group showed no upward trend compared with the DNR group; ↓↓, compared with the DNR group, the DHA27 group showed a statistically significant decrease; ↓, the DHA27 group showed a decreasing trend compared with the DNR group, but there was no statistically significant difference.

Supplementary Table 2

Effect of DHA27 antibacterial sensitization and tobramycin (TOB) resistance on 16S rRNA methyltransferase (16S-RMtases) in *Pseudomonas aeruginosa*

Isolate name	MIC		FICI	16S rRNA	Effect on 16S-RMtases	
	TOB	DHA27			TOB	TOB+DHA27
PA05	512	＞1024	＜0.13	*armA*	↑↑	↓
				rmtA	↑↑	↓↓
				rmtB	↑↑	↓↓
				rmtC	↓	↑↑
				rmtD	↑↑	↑
PA16	1024	＞1024	＜0.13	*armA*	↑↑	↓
				rmtA	↑↑	↓
				rmtB	×	×
				rmtC	↑	↓↓
				rmtD	↑↑	↓↓
				armA	↑↑	↓↓
				rmtA	↑↑	↓

				rmtB	↑↑	↑↑
PA01	1024	＞1024	＜0.19	*rmtC*	↑	↑
				rmtD	↑↑	↓↓
				armA	↑↑	↑↑
				rmtA	↑↑	↑↑
				rmtB	↑	↑↑
PA06	512	＞1024	＜0.19	*rmtC*	×	×
				rmtD	↑↑	↓
				armA	↑↑	↓
				rmtA	↑↑	↓↓
				rmtB	×	×
				rmtC	↓↓	↓
PA07	256	＞1024	＜0.19	*rmtD*	↑↑	↑
				armA	↑↑	↑↑
				rmtA	↑	↑
				rmtB	↑↑	↑↑

				rmtC	↓↓	↑
PA08	256	＞1024	＜0.19			
				rmtD	↑↑	↑
				armA	↑↑	↑
				rmtA	↑↑	↓↓
				rmtB	↑↑	↓
				rmtC	↑↑	↓
PA13	＞1024	＞1024	＜0.19			
				rmtD	↑↑	↓↓
				armA	↑↑	↑↑
				rmtA	↑↑	↑↑
				rmtB	↓↓	—
				rmtC	↓↓	↑↑
PA17	1024	＞1024	＜0.19			
				rmtD	↑	↑
				armA	↑↑	↑
				rmtA	↑↑	↑
				rmtB	↑↑	↓
				rmtC	↑↑	↑
PA04	512	＞1024	＜0.25			
				rmtD	↑↑	↑
				armA	↑↑	↑

				rmtA	↑	↑↑
				rmtB	×	×
PA09	1024	＞1024	＜0.25	*rmtC*	↓	↓
				rmtD	↑	↑↑
				armA	↓	↑↑
				rmtA	↓↓	↑↑
				rmtB	×	×
				rmtC	↓↓	↑↑
PA02	1024	＞1024	＜0.31	*rmtD*	↓↓	↑↑
				armA	↓↓	↓↓
				rmtA	↓	↓
				rmtB	↓↓	↓↓
PA19	1024	＞1024	＜0.31	*rmtC*	×	×
				rmtD	↓	↓
				armA	↑	↓↓
				rmtA	↑↑	↓
				rmtB	×	×
				rmtC	↑↑	↑

PA20	1024	＞1024	＜0.38	*rmtD*	↑↑	↓

FICI, fractional inhibitory concentration index

(1) ↓↓, Compared with the TOB group, the drug combination group showed a statistically significant difference; ↓, the drug combination group showed a downward trend compared with the TOB group, but there was no statistically significant difference; -, compared with the TOB group, the drug combination group showed no downward trend; ↑, the drug combination group showed an upward trend compared with the TOB group, but there was no statistically significant difference; ↑↑, compared with the TOB group, the drug combination group showed a statistically significant upward trend; ×, some results did not come out:

(2) ↓↓, Compared with the broth group, the TOB group showed a statistically significant decrease; ↓, the TOB group showed a decreasing trend compared with the broth group, but there was no statistically significant difference; -, compared with the broth group, the TOB group showed no downward trend; ↑, the TOB group showed an upward trend compared with the broth group, but there was no statistically significant difference; ↑↑, compared with the broth group, the TOB group showed a statistically significant upward trend; ×, some results did not come out

Supplementary Table 3

Effect of DHA27 antibacterial sensitization and tobramycin (TOB) resistance on the mRNA expression of aminoglycoside-modifying enzymes (AMEs) in *Pseudomonas aeruginosa*

strain name	MIC		FICI	AMEs	Drug effects on AMEs	
	TOB	DHA27			TOB	TOB+DHA27
PA05	512	＞1024	＜0.13	*aac(3)-I*	↑↑	↓
				aac(3)-II	↑	↓
				aac(3)-IV	↑↑	↓↓
				aac(6')-Ib	↑↑	↓↓
				aac(6')-II	↑↑	↓↓
				ant(2")-Ia	↑↑	↓↓
				ant(2")-I	↑	↑
				ant(3")-I	↑↑	↑
				aph(3')-Ia	↑↑	↓
PA16	1024	＞1024	＜0.13	*aac(3)-I*	↑	↓
				aac(3)-II	↑↑	↓
				aac(3)-IV	↑↑	↓↓
				aac(6')-Ib	↑↑	↓
				aac(6')-II	↑↑	↓↓
				ant(2")-Ia	↑↑	↓
				ant(2")-I	↑↑	↓
				ant(3")-I	↑	↑↑
				aph(3')-Ia	↑↑	↑
PA01	1024	＞1024	＜0.19	*aac(3)-I*	↑↑	—
				aac(3)-II	↑↑	↓↓
				aac(3)-IV	↑↑	↓↓
				aac(6')-Ib	↓	↓↓
				aac(6')-II	—	↓
				ant(2")-Ia	↑↑	↓
				ant(2")-I	↑↑	↓↓
				ant(3")-I	↑↑	↓

				aph(3')-Ia	×	×
				aac(3)-I	↓	↓
				aac(3)-II	↓	↑
				aac(3)-IV	↑	↓
				aac(6')-Ib	↓	—
PA06	512	＞1024	＜0.19	*aac(6')-II*	↓	↓
				ant(2")-Ia	↑ ↑	↑
				ant(2")-I	↑	↓
				ant(3")-I	↑ ↑	↓ ↓
				aph(3')-Ia	↑	↑
				aac(3)-I	↑	↑
				aac(3)-II	↑	↑
				aac(3)-IV	↑ ↑	↓
				aac(6')-Ib	↑ ↑	↓
				aac(6')-II	↓ ↓	↓
PA07	256	＞1024	＜0.19	*ant(2")-Ia*	↑ ↑	↓ ↓
				ant(2")-I	↓	↑
				ant(3")-I	↑	↑
				aph(3')-Ia	×	×
				aac(3)-I	↑	↑ ↑
				aac(3)-II	—	↑
				aac(3)-IV	↑	—
				aac(6')-Ib	↓	↑
PA08	256	＞1024	＜0.19	*aac(6')-II*	↓ ↓	↑ ↑
				ant(2")-Ia	↑ ↑	↑ ↑
				ant(2")-I	↓ ↓	↑ ↑
				ant(3")-I	↑	↑
				aph(3')-Ia	↑	↓
				aac(3)-I	↑ ↑	↓ ↓
				aac(3)-II	↑ ↑	↓ ↓
				aac(3)-IV	↑	↓
				aac(6')-Ib	↑ ↑	↓ ↓
PA13	＞1024	＞1024	＜0.19	*aac(6')-II*	↑ ↑	↓
				ant(2")-Ia	↑ ↑	↓ ↓
				ant(2")-I	↑ ↑	↓ ↓
				ant(3")-I	↑ ↑	↓

				aph(3')-Ia	↑ ↑	↑
				aac(3)-I	↑	—
				aac(3)-II	↑	↑
				aac(3)-IV	↑ ↑	↑
				aac(6')-Ib	↑ ↑	↑
PA17	1024	＞1024	＜0.19	*aac(6')-II*	↑ ↑	↑
				ant(2")-Ia	↑ ↑	↑ ↑
				ant(2")-I	↑	↑
				ant(3")-I	↑ ↑	↑
				aph(3')-Ia	↑	↓
				aac(3)-I	—	↑
				aac(3)-II	↑ ↑	—
				aac(3)-IV	↑ ↑	—
				aac(6')-Ib	↑ ↑	↑ ↑
PA04	512	＞1024	＜0.25	*aac(6')-II*	↑ ↑	↓
				ant(2")-Ia	↑ ↑	↑
				ant(2")-I	↑	↓
				ant(3")-I	↑ ↑	↓
				aph(3')-Ia	↑ ↑	↑
				aac(3)-I	↑ ↑	↑ ↑
				aac(3)-II	↑ ↑	↓
				aac(3)-IV	↓	↑
				aac(6')-Ib	↓	↑ ↑
PA09	1024	＞1024	＜0.25	*aac(6')-II*	↓	↑
				ant(2")-Ia	↑	↑ ↑
				ant(2")-I	↓ ↓	↑ ↑
				ant(3")-I	↑	↓
				aph(3')-Ia	↑ ↑	↑ ↑
				aac(3)-I	↑ ↑	↑ ↑
				aac(3)-II	↓	—
				aac(3)-IV	↑ ↑	↑
				aac(6')-Ib	↓ ↓	↓
PA02	1024	＞1024	＜0.31	*aac(6')-II*	↑ ↑	—
				ant(2")-Ia	↓ ↓	↑ ↑
				ant(2")-I	↓ ↓	↓
				ant(3")-I	—	↑ ↑

				aph(3')-Ia	↑	↑
PA19	1024	＞1024	＜0.31	*aac(3)-I*	↓↓	↓↓
				aac(3)-II	↓	↑
				aac(3)-IV	↑↑	↓↓
				aac(6')-Ib	↓↓	↓↓
				aac(6')-II	↑	↓
				ant(2")-Ia	↓↓	↓↓
				ant(2")-I	—	—
				ant(3")-I	↓	↓
				aph(3')-Ia	↑	↓
PA20	1024	＞1024	＜0.38	*aac(3)-I*	↑↑	↓
				aac(3)-II	↑↑	↑
				aac(3)-IV	↑↑	—
				aac(6')-Ib	↑↑	↓
				aac(6')-II	↑↑	↓
				ant(2")-Ia	↑↑	↓↓
				ant(2")-I	↑↑	↓
				ant(3")-I	↑↑	↑↑
				aph(3')-Ia	↑↑	↑

(1) ↓↓, Compared with the TOB group, the drug combination group showed a statistically significant difference; ↓, the drug combination group showed a downward trend compared with the TOB group, but there was no statistically significant difference; -, compared with the TOB group, the drug combination group showed no downward trend; ↑, the drug combination group showed an upward trend compared with the TOB group, but there was no statistically significant difference; ↑↑, compared with the TOB group, the drug combination group showed a statistically significant upward trend; ×, some results did not come out.

(2) ↓↓, Compared with the broth group, the TOB group showed a statistically significant decrease; ↓, the TOB group showed a decreasing trend compared with the broth group, but there was no statistically significant difference; -, compared with the broth group, the TOB group showed no downward trend; ↑, the TOB group showed an upward trend compared with the broth group,

but there was no statistically significant difference; ↑↑, compared with the broth group, the TOB group showed a statistically significant upward trend; ×, some results did not come out.
